# Two Monogenetic Disorders, Activated PI3-Kinase-δ Syndrome 2 and Smith–Magenis Syndrome, in One Patient: Case Report and a Literature Review of Neurodevelopmental Impact in Primary Immunodeficiencies Associated With Disturbed PI3K Signaling

**DOI:** 10.3389/fped.2021.688022

**Published:** 2021-06-24

**Authors:** Nidia Moreno-Corona, Loïc Chentout, Lucie Poggi, Romane Thouenon, Cecile Masson, Melanie Parisot, Lou Le Mouel, Capucine Picard, Isabelle André, Marina Cavazzana, Laurence Perrin, Anne Durandy, Saba Azarnoush, Sven Kracker

**Affiliations:** ^1^Université de Paris, Imagine Institute, Paris, France; ^2^Laboratory of Human Lymphohematopoiesis, INSERM UMR 1163, Paris, France; ^3^Paris-Descartes Bioinformatics Platform, Imagine Institute, Paris, France; ^4^Genomics Core Facility, Institut Imagine-Structure Fédérative de Recherche Necker, INSERM U1163 et INSERM US24/CNRS UMS3633, Université de Paris, Paris, France; ^5^Hospital Robert Debré, Pediatric Immune-Hematology Service, Paris, France; ^6^Necker Hospital, Pediatric Hematology-Immunology and Rheumatology Unit, Assistance publique des hôpitaux de Paris (APHP), Paris, France; ^7^Paris Hospital, Study Center for Primary Immunodeficiencies—APHP, Paris, France; ^8^Necker Hospital, Biotherapy and Clinical Investigation Centre—APHP, Paris, France

**Keywords:** APDS2, PI3K signaling, PIK3R1, primary immunodeficiency, neurodevelopmental impact

## Abstract

Activated PI3-kinase-δ syndrome 2 (APDS2) is caused by autosomal dominant mutations in the *PIK3R1* gene encoding the p85α, p55α, and p50α regulatory subunits. Most diagnosed APDS2 patients carry mutations affecting either the splice donor or splice acceptor sites of exon 11 of the *PIK3R1* gene responsible for an alternative splice product and a shortened protein. The clinical presentation of APDS2 patients is highly variable, ranging from mild to profound combined immunodeficiency features as massive lymphoproliferation, increased susceptibility to bacterial and viral infections, bronchiectasis, autoimmune manifestations, and occurrence of cancer. Non-immunological features such as growth retardation and neurodevelopmental delay have been reported for APDS2 patients. Here, we describe a patient suffering from an APDS2 associated with a Smith–Magenis syndrome (SMS), a complex genetic disorder affecting, among others, neurological manifestations and review the literature describing neurodevelopmental impacts in APDS2 and other PIDs/monogenetic disorders associated with dysregulated PI3K signaling.

## Introduction

Class IA PI3 kinase (PI3K) are heterodimeric enzymes composed of a p110 catalytic subunit and a regulatory subunit. Their function is to convert phosphatidylinositol 4,5-bisphosphate (PIP2) to phosphatidylinositol 4,5-trisphosphate (PIP3), a reaction reversed by the PTEN phosphatase (1). PIP3 is an important lipid second messenger promoting activation of downstream signaling molecules such as AKT/protein kinase B and mTOR. The class IA PI3K catalytic subunits p110α, p110β, and p110δ are encoded by the genes *PIK3CA, PIK3CB*, and *PIK3CD*, respectively. The regulatory subunits p85α, p55α, and p50α are encoded by *PIK3R1*, whereas p85β and p55γ are encoded by *PIK3R2* and *PIK3R3*, respectively. P110δ is predominantly expressed in cells of the hematopoietic linage in contrast to the ubiquitously expressed p110α and p110β. Activated PI3Kδ signaling due to either autosomal dominant gain-of-function mutation in the *PIK3CD* gene or autosomal dominant loss-of-function mutation in the *PIK3R1* gene causes activated PI3-kinase-δ syndrome [APDS; referred as type 1 APDS (APDS1) and type 2 APDS (APDS2), respectively] (2, 3). Clinical presentation for both types of APDS patients are very similar, ranging from profound combined immunodeficiency (associated with lymphoproliferation, severe bacterial and viral infections from childhood) to isolated humoral defects (4, 5).

The vast majority of disease-causing APDS2 mutations affect the splice donor or splice acceptor sites of exon 11, leading to an alternative splice product in which exon 11 is deleted (4, 6–9), enabling the expression of a shortened mutant p85α (and p50α and p55α) protein lacking part of the iSH2 domain (Δ434_475) (3, 10). The mutant protein p85α^Δ434_475^ particularly disturbs the regulation of p110δ, resulting in increased p110δ signaling in APDS2 patients lymphocytes (3, 10). Hydrogen–deuterium exchange mass spectrometry analysis provides a structural explanation why APDS2 resembles APDS1 (11): the inhibitory interactions of the nSH2, iSH2, and cSH2 domains are especially disrupted within the p85α^Δ434_475^/*p*110δ complex in contrast to only mild disturbance within the p85α^Δ434_475^/*p*110α complex. A missense *PIK3R1* p.N564K variant causing APDS2 has been reported (12), suggesting that also missense variants can have different impacts on p85α/p110δ versus p85α/p110α complexes. Of note, the same *PIK3R1* p.N564K variant has been identified in a patient belonging to a cohort of patients presenting with macrocephaly and intellectual disability (13). Growth impairment (−2 standard deviations of height) was especially noted in APDS2 patients (4). In a cohort study of APDS2 patients, 14 (45%) of 31 patients showed growth impairments affecting height and weight similarly as body mass indices were within normal ranges in all but two patients (4). Neurodevelopmental delay (global developmental or isolated speech delay) was recognized in both types of APDS (19 and 32% for APDS1 and APDS2, respectively) (4, 5). Moreover, both autism spectrum disorders and macrocrania have been reported in APDS cohorts (14). Three further patients were described as affected by anxiety disorders, with a diagnosis of autism, and three children were reviewed by psychological services for behavioral issues (4, 5).

Overall, a large spectrum of clinical features, including non-immunological ones, affects both types of APDS. The great heterogeneity observed on a patient-to-patient comparison suggests that environmental factors, among them history of infections with different pathogens, microbiota, and/or genetic “modifying” factor(s), contribute to the disease presentation.

Smith–Magenis syndrome (SMS) is a complex genetic disorder characterized by intellectual disability, sleep disturbances, and distinct craniofacial and skeletal anomalies (15, 16). SMS is caused by the retinoic acid–induced 1 (*RAI1*) haploinsufficiency. Approximately 90% of SMS cases carry a deletion of a 17p11.2 region encompassing multiple genes and including the *RAI1* gene locus. *RAI1* contains six exons, four of which are protein coding. Approximately 10% of all the SMS patients carry heterozygous mutations within the *RAI1* coding region (15). Mutations reported to cause SMS include premature stop codons and frameshift mutations (small deletions or insertions) (15–18).

## Case Report

**Figure 1:**
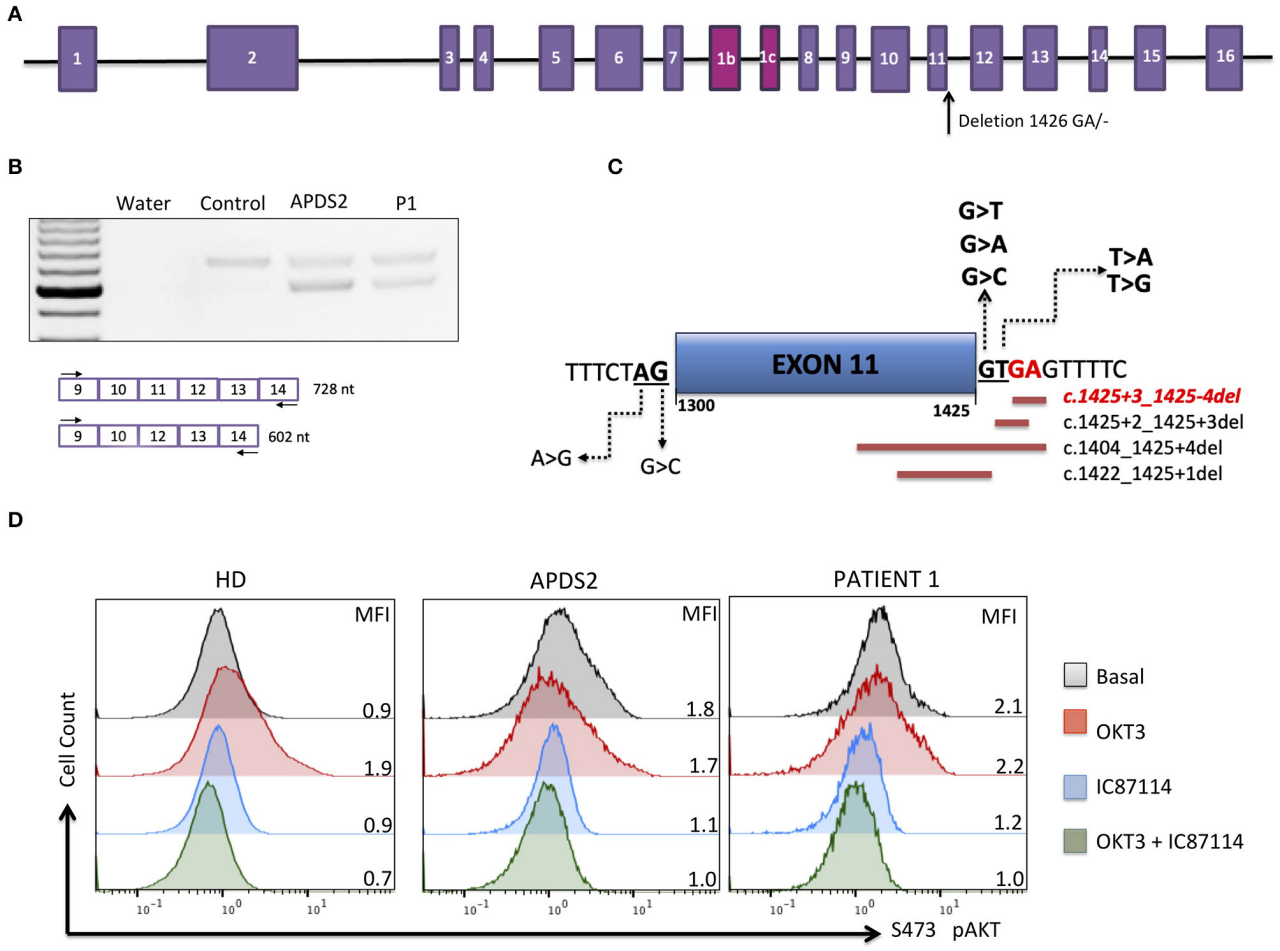
Molecular APDS2 characteristics. **(A)** Schematic representation of the *PIK3R1* gene and illustrated localization of identified mutation. **(B)** RT-PCR of PIK3R1 mRNA from T-cell blast of a control, a patient with APDS2 (3), and the investigated patient. **(C)** Schematic representation of mutations affecting splicing of exon 11 annotated in ClinVAR. In red is the mutation of investigated patient. **(D)** AKT phosphorylation analysis in T-cell blasts (cultured for 13 days) from a healthy control, an APDS2 patient, and the investigated patient. The analysis was performed as described by Deau et al. (3). Mean fluorescence intensity (MFI) is indicated for each sample.

The patient was born at normal term and good newborn mensuration to unrelated parents from North African origin. She was the last child of three siblings. Her two brothers have been monitored for asthma. No family history of genetic disorders or young death has been reported. The first year of life was characterized by repeated urinary tract infections (three episodes of pyelonephritis), resulting in the discovery of urinary tract malformation (duplication of left ureter), resulting in a solitary functional kidney and vesicoureteral reflux requiring a pyeloureteral nephrectomy before the age of 1 year. She also presented with recurrent ear infections (otitis media) and three episodes of pneumonia with gastroesophageal reflux. Tympanocentesis (tympanic membranes incision) was performed because of recurrent otitis media and otorrhea at the age of 2 years. An adenoidectomy was also performed at this age. Intravenous immunoglobulin (Ig) replacement therapy was started at the age of 3 years as increased IgM associated with decreased IgG and normal IgA serum levels were detected (Table 1). Although under Ig replacement therapy the patient continued to suffer from recurrent (chronic) otitis media associated with *Streptococcus haemolyticus* A and *Staphylococcus* infections. She also suffered from a *Staphylococcus aureus* Meti S infection causing sepsis at the age of 11.5 years. The patient developed progressive neurodevelopmental disorders with delayed acquisition of walking at 24 months of life, delayed language development, and becoming unintelligible after 4 years old. In parallel, she presented with growth disorders evolving regularly on +0.5 SD for weight and −1 SD for height until the age of 1 year. Then, a break in the stature curve appeared until −3 SD at the age of 3 years, associated with a rapid onset of obesity, becoming severe from age of 6 years. Clinical examination revealed morphologic abnormalities including hypertelorism, strabismic amblyopia, large philtrum, genu valgum and adipomastia, and symptoms of lymphoproliferation presenting as hepatosplenomegaly associated with upper centimetric lymphadenopathies. Lymphoproliferation symptoms disappeared at the age of 7 years. Vascular malformation with carotid stenosis and moyamoya was found using cerebral magnetic resonance imaging. Over the years, the patient developed behavior disorders requiring neuroleptic medicines, pedopsychiatric monitoring and institutionalization, sleep apnea syndrome requiring an equipment with non-invasive nocturnal ventilation, and hyperandrogenia with primitive amenorrhea or polycystic ovary syndrome. The etiologic investigation including karyotypic, array comparative genomic hybridization, and genetic analysis was negative. Panel sequencing of genes implicated in intellectual deficiencies identified a non-described *de novo* heterozygous non-sense mutation of *RAI1* gene c.2701A>T p.Lys901^*^ not found in the two parents' blood tests, responsible for an SMS. However, the whole phenotype could not be explained by this syndrome, and an inborn error of immunity was suspected because of the infection history, the hypogammaglobulinemia and immune phenotyping of the patient indicating B-cell lymphopenia associated with an increased frequency of transitional B cells, a decreased frequency of naive (CD45RA^+^) and recent thymic emigrants (CD45RA^+^CD31^+^) CD4 and naive (CD45RA^+^CCR7^+^) CD8 T-cell subsets, increased frequency of CD8 (CCR7^−^*CD*45*RA*^−^ and CCR7^−^*CD*45*RA*^+^) T-cell subsets, and an inverted CD4/CD8 T-cell ratio (Table 1). Whole-exome sequencing of DNA from the patient and both parents was performed on a research basis. Filtering of annotated variants after a strict *de novo* genetic model confirmed the non-sense mutation of *RAI1* and showed another *de novo* variant (2 nucleotide deletion) located within the intronic splice region (splice donor site) of the *PIK3R1* gene at position GRCh37/hg19; chr5: 67589664; c.1425+3delGA, giving evidence for an APDS2. Analysis of patients' derived T-cell blast mRNA indicated exon skipping of coding exon11 (Figures 1A–C). Increased phosphorylation of AKT/protein kinase B at position Ser473 was observed in patients' T-cell blasts vs. healthy control T-cell blasts (Figure 1D). Treatment with a p110 δ-specific inhibitor (IC87114) abrogated those differences, indicating that increased PI3K δ-signaling at basal level was responsible for the high level of AKT phosphorylation at Ser473 (Figure 1D). Together, our functional analysis demonstrated that the *de novo* c.1425+3delGA mutation causes exon skipping of exon 11 and subsequent activation of PI3K δ-signaling in lymphocytes. Since the discovery of APDS2, administration of immunomodulatory agents such as rapamycin and p110δ-specific inhibitor has been under consideration.

**Table 1:** Immunological characteristics of the patient.

	**Patient**	**Reference values**
Age at evaluation (years)	16	
Lymphocytes (/μl)	2,573	1,849–2,788
Natural killer cells (CD16+CD56+) (/μl)	387	70–480
T cells (CD3+) (/μl)	2,213	1,000–2,200
CD4 T cells (/μl)	515	530–1,300
CD8 T cells (/μl)	1,570	330–920
Naive CD4 T cells (CD45RA+/CD4+) (%)	35	58–70
Naive CD4 recent thymic emigrants T cells (CD31+CD45RA+/CD4+)	19	43–55
Naive CD8 T cells (CCR7+CD45RA+CD8+) (%)	4	52–68
Central memory CD8 T cells (CCR7+CD45RA–/CD8+) (%)	2.5	3–4
Effector memory CD8 T cells (CCR7–CD45RA–/CD8+) (%)	35	11–20
Terminal differentiating effector memory CD8 T cells (CCR7–CD45RA+/CD8+) (%)	58.5	16–28
B cells CD19 (/μl)	48	183–628
Transitional B cells (CD24++CD38++/CD19+) (%)	33	<11
Age at evaluation (years)	2	16
IgG (g/L)	<0.33 (3.7–15.8)	10.09^*^ (6–16)
IgA (g/L)	0.81 (0.3–1.3)	2.07 (0.8–2.8)
IgM (g/L)	2.73 (0.5–2.2)	6.14 (0.5–1.9)

* *Under Ig replacement; age-matched Ig reference values in brackets*.

## Discussion

Our patient presents clinically with two separable diseases: APDS2, caused by a novel *de novo PIK3R1* splice donor site mutation, and SMS, caused by a novel *de novo RAI1* non-sense mutation. Many aspects of disease manifestations can be affiliated to one or the other disease. Although intellectual disabilities, behavior problems, and growth retardation in the patient presented here are likely triggered by SMS, it is important to note that growth retardation and global developmental delay has been reported for several APDS2 patients, making it difficult to untangle these aspects with certainty (Table 2). Studies of primary immunodeficient patients with either autosomal dominant, autosomal recessive, or compound heterozygous variants in *PIK3R1* and *PIK3CD* genes emphasized the importance of a strict balance of PI3Kδ signaling for optimal immune responses (19–24). However, expression of p110δ in the murine brain (5) and reported neurodevelopmental delay in both types of APDS patients suggested that balanced PI3Kδ signaling is also important for neurodevelopment (Table 3). This is further supported by a recent study assessing cognitive functions of two APDS1 patients (one of them diagnosed with psychomotor developmental delay and autism spectrum disorder) and a murine APDS1 model (E1020K knock-in), indicating that APDS1 patients presented with visuomotor deficits and that p110δ^E1020K^ mice exhibited impairments in motor behavior, learning, and repetitive behavior patterning (25).

**Table 2:** Clinical features of APDS2, SMS, and reported patient.

	**APDS2**	**SMS**	**Case report patient**
**Infectious complications**			
Upper respiratory infections	X		X
Chronic otitis	X	X	X
Pneumonia	X		X
Sepsis	X^*^		X
Urinary tract infections/pyelonephritis			X
**Adenopathy**			
Lymphadenopathy	X		X
Splenomegaly	X		X
Hepatomegaly	X	X	X
**Neurological/behavioral**			
Behavioral problems	X	X	X
Neurodevelopmental delay	X	X	X
Variable mental retardation		X	X
Speech delay	X	X	X
Sleep disturbance		X	X
Skeletal/craniofacial			
Short stature	X	X	X
**Ocular abnormalities**			
Strabismus		X	X
**Other features**			
Vascular abnormalities (moyamoya)		X	X
Renal/urinary tract abnormalities		X	X

* *Two reports*.

**Table 3:** Cases of neurodevelopmental delay in inborn errors of immunity/monogenic deficiencies associated with disturbed class 1A PI3K signaling.

**Disease**	**OMIM#**	**Gene**	**Inheritance**	**PI3K signaling**	**Immunological defect**	**Neurodevelopmental delay**	**References**
APDS1	615513	PIK3CD	AD	Increased	Ig serum level, B and T	19% of patients in cohort study;global development, speech delay, autism spectrum disorder	(5, 25)
APDS2	616005	PIK3R1	AD	Increased	Ig serum level, B and T	31% of patients in cohort study; cognitive impairments, learning disabilities	(4)
SHORT syndrome	269880	PIK3R1	AD	Decreased	N.R.	Behavioral problemsspeech delay	(26, 27)
p85α deficiency	615214	PIK3R1	AR	N.R.	B absent, Ig serum level,	N.R.	(23)
P110delta deficiency		PIK3CD	AR	Decreased	B and NK decreased, T impaired Ig serum level,	N.R.	(20, 22, 24)
Roifman–Chitayat syndrome	613328	PIK3CD/*KNSTRN*	AR		Ig serum level, B and NK decreased, T impaired	Developmental delay; cognitive, speech, and motor retardation,tremor, ataxia	(19)
APDS-L/Cowden syndrome1/macrocephaly/autism syndrome	158350/605309	PTEN	AD	Increased	Ig serum level, B and T	Autism spectrum disorders; developmental delay and macrocephaly	(28–32)

*NK, natural killer; N.R., not reported*.

Macrocephaly, developmental delay, autism spectrum disorders in addition to an increased risk of cancers (mainly breast, thyroid, and endometrium cancer), benign tumors (hamartomas), and immunodeficiency predisposing patients to APDS-like clinical manifestations (e.g., increased susceptibility to bacterial infections, bronchiectasis and lymphadenopathy including hepatosplenomegaly) are characteristics for a variety of rare syndromes caused by heterozygous loss-of-function germline mutations in the *phosphatase and tensin homolog deleted on chromosome 10 (PTEN)* gene [Cowden syndrome, Bannayan–Riley–Ruvalcaba syndrome, proteus syndrome, and Proteus-like syndrome, PTEN hamartoma tumor syndrome (PHTS), APDS-L; OMIM: # 158350] (28–32), further indicating a detrimental effect of increased PI3K signaling for neurodevelopment. A retrospective cohort study of pediatric patients diagnosed with autism spectrum disorders or developmental delay and macrocephaly indicated a *PTEN* mutation prevalence of 8 and 12%, respectively, pinpointing the frequency of this genetic defect in these neurological diseases (33).

Decreased PI3K signaling might also impair neurodevelopment. Autosomal dominant loss-of-function *PIK3R1* mutations leading to decreased PI3K signaling activity cause a rare genetic condition called SHORT syndrome. The acronym SHORT stands for typical clinical features of this disease as: **s**hort stature, **h**yperextensibility of the joints and/or inguinal hernias, **o**cular depression (deep-set eyes), **R**ieger anomaly, and **d**elayed teething (26). Of note, several patients diagnosed with SHORT syndrome presented with delay speech development (26, 27, 34) and behavioral problems (34). No immunological abnormality has been reported so far. Two different autosomal recessive *PIK3R1* non-sense mutations causing p85α deficiency have been described to impair B-cell development and to cause agammaglobulinemia (23, 24). Although only very rare patients have been described (three up to now), there is no evidence for associated neurodevelopmental abnormalities. In two patients of a familial case diagnosed with Roifman–Chitayat syndrome caused by the combination of two gene defects: p110 δ deficiencies (homozygous non-sense mutation in *PIK3CD*) and small kinetochore-associated protein (SKAP) deficiency (frameshift homozygous mutation in *KNSTRN*) developmental delay presenting as either significant cognitive, speech, and motor retardation or global developmental delay, tremor, and ataxia (diagnosed for both patients early in life) were reported (19). As neurodevelopmental manifestations were not reported in several familial cases of p110δ deficiencies caused by biallelic non-sense, frameshift, or loss-of-function mutations in *PIK3CD* (20, 22, 35), it is likely that the SKAP deficiency, or possibly the combination of the two gene defects, is responsible for the neurological features observed in these two Roifman–Chitayat patients.

## Conclusion

Here, we described a patient with a complex clinical presentation, carrying a novel (*de novo*) donor splice site mutation in the *PIK3R1* gene and a novel (*de novo*) non-sense mutation in the *RAI1* gene. This is the first time that the clinical and immunological phenotype of an APDS2 patient presenting with two independent monogenetic disorders, APDS2 and SMS, has been described. Cohort studies of both types of APDS indicated a large spectrum of clinical presentation from very mildly affected (or even an asymptomatic) to severe combined immunodeficient patients. Neurological development appears to be variable in both types of APDS from inconspicuous to autism spectrum disorders. This variable clinical spectrum of immunological and neurological manifestations could be explained by individual patient-by-patient dependent environmental, epigenetic, and genetic factors. Our study provides a further example of an unusual clinical presentation of APDS due to another associated gene defect.

## Data Availability Statement

The original contributions presented in the study are included in the article/supplementary material, further inquiries can be directed to the corresponding author/s.

## Ethics Statement

The studies involving human participants were reviewed and approved by Comité de Protection des Personnes Ile de France II, Paris, France; reference no. CPP:2015-01-05. Written informed consent to participate in this study was provided by the participants' legal guardian/next of kin. Written informed consent was obtained from the minor(s)' legal guardian/next of kin for the publication of any potentially identifiable images or data included in this article.

## Author Contributions

NM-C, AD, SA, and SK wrote the manuscript. CP, LLM, LPe, and SA provided clinical care and collected clinical data. NM-C, LC, LPo, RT, CM, and MP performed experiments. SK supervised and contributed to the whole work. All authors analyzed data and agreed to the manuscript.

## Conflict of Interest

SK reports grants and payments for service agreements and travel from UCB Pharma and is a designated inventor on published patent application WO2017/198590. The remaining authors declare that the research was conducted in the absence of any commercial or financial relationships that could be construed as a potential conflict of interest.
